# Tris(2,6-dibenzoyl-4-methyl­phenolato-κ^2^ *O* ^1^,*O* ^2^)cobalt(III)

**DOI:** 10.1107/S1600536814001664

**Published:** 2014-01-29

**Authors:** Abhishek K. Gupta, Sanjay Srivastava, Ray J. Butcher

**Affiliations:** a Department of Material Science and Metallurgical Engineering, Maulana Azad National Institute of Technology, Bhopal 462 051, India; b Department of Chemistry, Howard University, 525 College Street NW, Washington, DC 20059, USA

## Abstract

In the title compound, [Co(C_21_H_15_O_3_)_3_], the Co^III^ ion is coordinated in a slightly distorted octa­hedral environment by three phenolate O and three benzoyl O atoms from three monoanionic bidentate 2,6-dibenzoyl-4-methyl­phenolate ligands. The dihedral angles between the mean planes of the central phenolate rings and the peripheral phenyl rings are 46.62 (10)/87.06 (9), 60.44 (8)/23.13 (8) and 46.49 (6)/65.29 (6)°. The crystal packing is stabilized by weak inter­molecular C—H⋯O inter­actions. Mol­ecules are further linked by two π–π [centroid–centroid distances = 3.8612 (14) and 3.9479 (14) Å] and four C—H⋯π inter­actions, forming a three-dimensional network.

## Related literature   

For phenol-based diketones, see: Gupta *et al.* (2002 ▶, 2012*a* ▶). For material and biological applications, see: Church & Halvorson (1959 ▶); Olsson *et al.* (2005 ▶); Burschka *et al.* (2013 ▶); Erkkila *et al.* (1999 ▶); Metcalfe & Thomas (2003 ▶); Generex & Barton (2010 ▶). For related structures, see: Gupta *et al.* (2012*b* ▶); Huang *et al.* (2013 ▶).
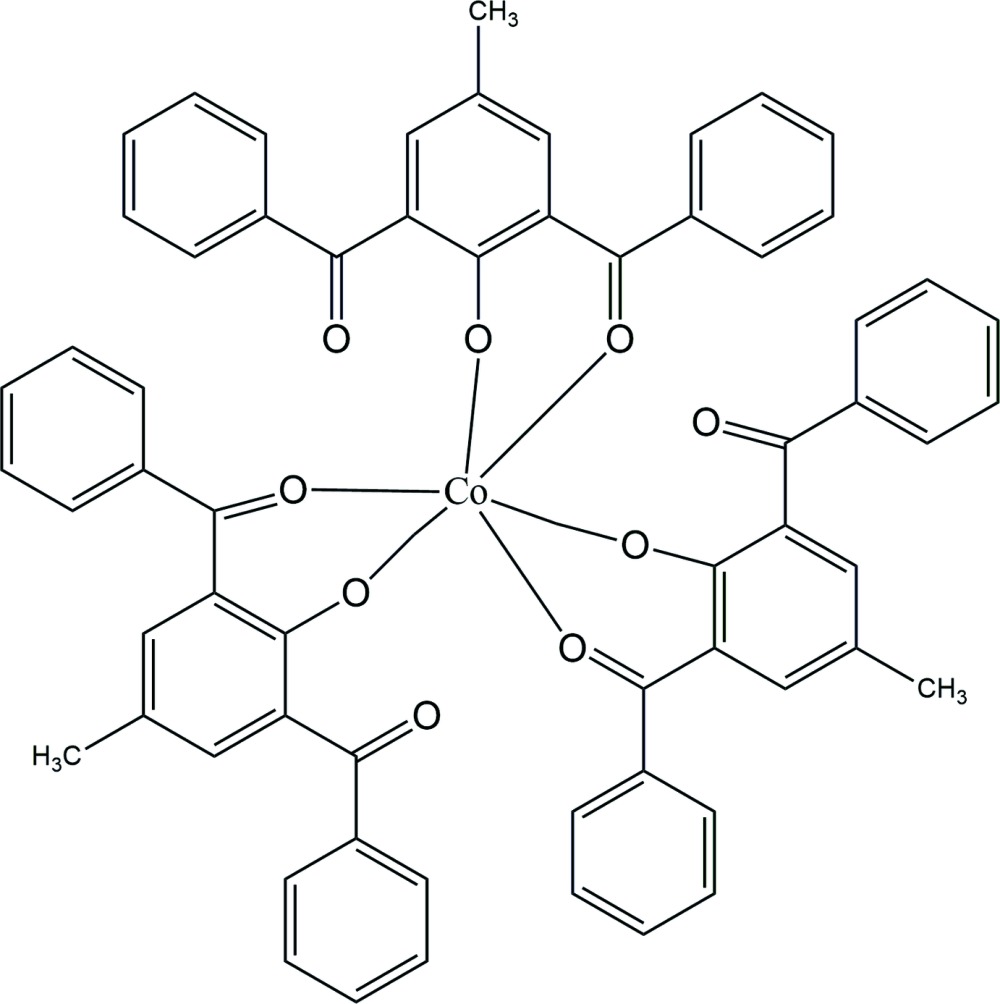


## Experimental   

### 

#### Crystal data  

[Co(C_21_H_15_O_3_)_3_]
*M* *_r_* = 1004.92
Monoclinic, 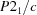

*a* = 11.2858 (3) Å
*b* = 17.5442 (4) Å
*c* = 24.7745 (5) Å
β = 92.8922 (19)°
*V* = 4899.12 (19) Å^3^
*Z* = 4
Cu *K*α radiation
μ = 3.25 mm^−1^
*T* = 123 K
0.46 × 0.18 × 0.15 mm

#### Data collection  

Agilent Xcalibur (Ruby, Gemini) diffractometer
Absorption correction: analytical [*CrysAlis PRO* (Agilent, 2012 ▶), based on expressions derived by Clark & Reid (1995 ▶)] *T*_min_ = 0.477, *T*_max_ = 0.705
21885 measured reflections
10145 independent reflections
9001 reflections with *I* > 2σ(*I*)
*R*_int_ = 0.045

#### Refinement  

*R*[*F* ^2^ > 2σ(*F* ^2^)] = 0.055
*wR*(*F* ^2^) = 0.154
*S* = 1.06
10145 reflections
661 parameters
H-atom parameters constrained
Δρ_max_ = 0.51 e Å^−3^
Δρ_min_ = −0.62 e Å^−3^

### 

Data collection: *CrysAlis PRO* (Agilent, 2012 ▶); cell refinement: *CrysAlis PRO*; data reduction: *CrysAlis PRO*; program(s) used to solve structure: *SHELXS97* (Sheldrick, 2008 ▶); program(s) used to refine structure: *SHELXL97* (Sheldrick, 2008 ▶); molecular graphics: *SHELXTL* (Sheldrick, 2008 ▶); software used to prepare material for publication: *SHELXTL*.

## Supplementary Material

Crystal structure: contains datablock(s) I. DOI: 10.1107/S1600536814001664/hg5379sup1.cif

Structure factors: contains datablock(s) I. DOI: 10.1107/S1600536814001664/hg5379Isup2.hkl

CCDC reference:

Additional supporting information:  crystallographic information; 3D view; checkCIF report

## Figures and Tables

**Table 1:** Hydrogen-bond geometry (Å, °) *Cg*7, *Cg*9, *Cg*11 and *Cg*12 are the centroids of the C9*A*–C14*A*, C9*C*–C14*C*, C16*B*–C21*B* and C16*C*–C21*C* rings, respectively.

*D*—H⋯*A*	*D*—H	H⋯*A*	*D*⋯*A*	*D*—H⋯*A*
C12*A*—H12*A*⋯O2*B* ^i^	0.95	2.60	3.442 (3)	147
C13*A*—H13*A*⋯O3*C* ^i^	0.95	2.48	3.278 (3)	141
C13*B*—H13*B*⋯O3*B* ^ii^	0.95	2.39	3.311 (3)	162
C11*C*—H11*C*⋯O3*C* ^iii^	0.95	2.40	3.313 (3)	161
C10*B*—H10*B*⋯*Cg*12	0.95	2.70	3.634 (3)	166
C11*B*—H11*B*⋯*Cg*7^iv^	0.95	2.72	3.479 (3)	137
C18*C*—H18*C*⋯*Cg*9^iv^	0.95	2.99	3.720 (4)	135
C20*C*—H20*C*⋯*Cg*11^v^	0.95	2.88	3.332 (3)	110

Symmetry codes: (i) 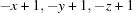; (ii) 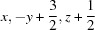; (iii) 
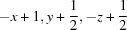
; (iv) 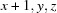; (v) 
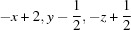
.

**Fig. 1.**
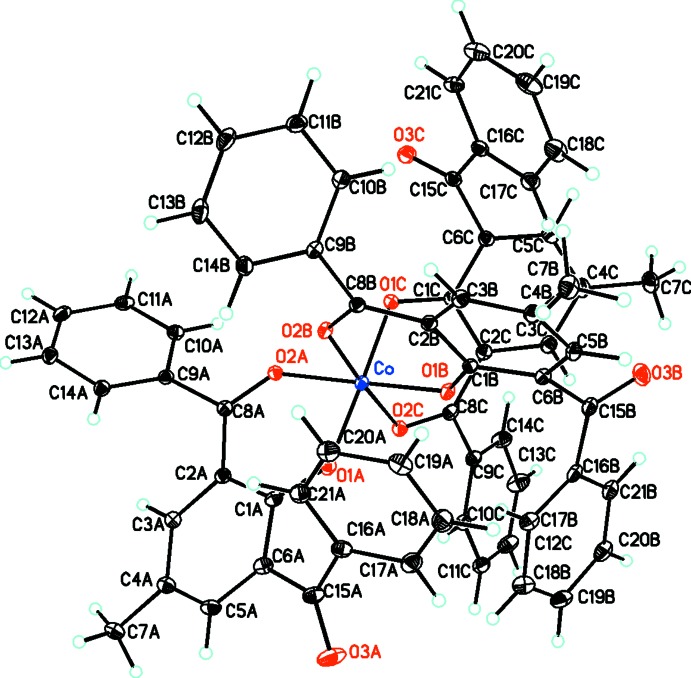
Molecular structure of the title compound showing atom numbering scheme. Displacement ellipsoids are drawn at the 50% probability level.

**Fig. 2.**
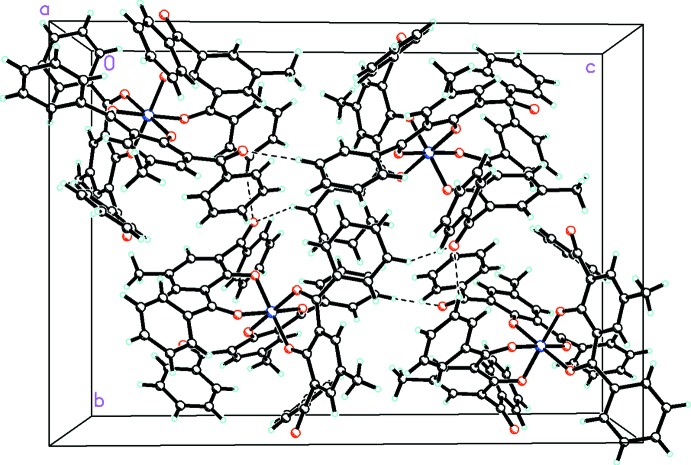
Packing diagram of [Co(C21H15O3)3] viewed along a axis. Dashes lines indicate a weak C–H···O intermolecular interactions.

## supplementary crystallographic information

### 1. Comment

In recent years phenol-based diketones have been widely used as ligands forming complexes with interesting properties that are useful in material science (Church & Halvorson, 1959; Olsson *et al.*, 2005; Burschka *et al.*, 2013) and in biological systems (Erkkila *et al.*, 1999; Metcalfe & Thomas, 2003; Generex & Barton, 2010). The crystal structure of 4-methyl-2,6-dibenzoylphenol (mdbpH)), 4-*tert*-butyl-2,6-dibenzoylphenol (bdbpH) and their chromium(III) complexes have been reported earlier (Gupta *et al.*, 2002, 2012*a*, 2012*b*). We herein report the synthesis and X-ray crystal structure analysis of the title compound.

The molecular structure of the title compound, [Co(C_21_H_15_O_3_)_3_], is shown in (Fig.1). The three monoanionic ligands (2,6-PhCO)_2_(4-Me)C_6_H_2_O^-^ are bidentate, coordinating through phenolic O and benzoyl O atoms to give a *mer*–CoO_3_O_3_ octahedral configuration. The coordination geometry around the Co(III) ion deviates slightly from an ideal octahedral coordination as evidenced by the *trans* angles, O1C/Co/O1A (178.32 (7)°), O2B/Co/O2C (176.88 (7)°) and O1B/Co/O2A (178.76 (7)°). The remaining angles vary between 87.01 (7)° and 93.25 (7)°, whereby the smallest values correspond to the O–Co–O bond angles in the three chelate rings, O1A/Co/O2A 88.47 (7)°, O1B/Co/O2B 90.02 (7)° and O1C/Co/O2C 87.01 (7)°. The Co–O (phenolic) distances [mean 1.932 Å] are similar and comparable to those reported for other mononuclear complexes, [Cr(mdbp)_3_, mean 1.931 Å] (Gupta *et al.*, 2012*b*) and [Co*L*_3_] (*L* = 4- bromo-2-(methyliminomethyl)phenolate) [mean 1.890 Å] (Huang *et al.*, 2013) but significantly shorter than the Co–O (benzoyl) distance [mean 1.974 Å]. The dihedral angles between the mean planes of the central phenolato rings (C1A–C6A; C1B–C6B; C1C–C6C) and the peripheral phenyl rings (C9A–C14A & C16A–C21A; C9B–C14B & C16B–C21B; C9C–C14C & C16C–C21C) are 46.62 (10)° & 87.06 (9)°; 60.44 (8)° & 23.13 (8)° and 46.49 (6)° & 65.29 (6)°, respectively, indicating that there is no conjugation between the phenolato and phenyl rings in the mdbp ligands. Further, there are significant differences in the O–C–C–C torsion angles, O1A/C1A/C2A/C8A (–9.9 (4)°), O1B/C1B/C2B/C8B (–2.1 (4)°) and O1C/C1C/C2C/C8C (3.8 (4)°) than that observed in the ligand, O1/C1/C2/C8 (0.0 (3)°) (Gupta *et al.*, 2002) which suggest that distortions are driven by steric interactions. The crystal packing is stabilized by weak C–H···O intermolecular interactions (Fig.2, Table 1). Molecules are further linked by two π–π [*Cg*2–*Cg*10 = 3.9479 (14) Å, *Cg*7—*Cg*7i = 3.8612 (14) Å, symmetry code (i): 1 - *x*, -*y*, -*z*, where *Cg*2, *Cg*7 and *Cg*10 are the centroids of the phenolate (Co/O1B/C1B/C2B/C8B/O2B), and phenyl (C9A–C14A, C16A–C21A) rings, respectively and four C–H···π (C10B–H10B–*Cg*12 = 3.634 (3) Å, C11B–H11B–*Cg*7i = 3.479 (3) Å, C18C–H18C–*Cg*9i = 3.720 (4) Å, C20C–H20C–*Cg*11ii = 3.332 (3) Å, symmetry code (i): 1 + *x*, *y*, *z*; ii: 2 - *x*, -1/2 + *y*, +1/2 - *z* where *Cg*9, *Cg*11 and cg12 are the centroids of phenyl (C9C–C14C, C16B–C21B, C16C–C21C rings)) interactions to form a three-dimensional network.

### 2. Experimental

An ethanolic solution of Co(ClO_4_)_2_.6H_2_O (0.366 g, 1.00 mmol) was added dropwise to the stirred hot solution of 2,6-dibenzoyl-4-methylphenol (0.948 g, 3.00 mmol) in ethanol under argon. The resulting wine-red solution was heated to reflux at 70–80 °C. The clear solution thus obtained was filtered and allowed to cool at ambient temperature. Slow evaporation of the solvent resulted in dark-brown prism-shaped crystals within a few days (yield: 0.80 g, 80%; m.p. 260–262 °C). Analysis calculated for C_63_H_45_O_9_Co (%): C 75.29, H 4.51; found: C 75.40, H 4.60.

### 3. Refinement

H atoms were positioned geometrically and refined using the riding model, with C–H distance of 0.95–0.98 Å, with *U_iso_* (H) = 1.20 *U_eq_* (C) or 1.50 *U*_eq_ (C) for methyl H atoms.

### Crystal data

**Table:**

[Co(C_21_H_15_O_3_)_3_]	*F*(000) = 2088
*M**_r_* = 1004.92	*D*_x_ = 1.362 Mg m^−^^3^
Monoclinic, *P*2_1_/*c*	Cu *K*α radiation, λ = 1.54184 Å
Hall symbol: -P 2ybc	Cell parameters from 9359 reflections
*a* = 11.2858 (3) Å	θ = 3.1–77.4°
*b* = 17.5442 (4) Å	µ = 3.25 mm^−^^1^
*c* = 24.7745 (5) Å	*T* = 123 K
β = 92.8922 (19)°	Prism, dark brown
*V* = 4899.12 (19) Å^3^	0.46 × 0.18 × 0.15 mm
*Z* = 4	

### Data collection

**Table:**

Agilent Xcalibur (Ruby, Gemini) diffractometer	10145 independent reflections
Radiation source: Enhance (Cu) X-ray Source	9001 reflections with *I* > 2σ(*I*)
Graphite monochromator	*R*_int_ = 0.045
Detector resolution: 10.5081 pixels mm^-1^	θ_max_ = 77.6°, θ_min_ = 3.6°
ω scans	*h* = −13→14
Absorption correction: analytical [*CrysAlis PRO* (Agilent, 2012), based on expressions derived by Clark & Reid (1995)]	*k* = −21→15
*T*_min_ = 0.477, *T*_max_ = 0.705	*l* = −31→24
21885 measured reflections	

### Refinement

**Table:**

Refinement on *F*^2^	Primary atom site location: structure-invariant direct methods
Least-squares matrix: full	Secondary atom site location: difference Fourier map
*R*[*F*^2^ > 2σ(*F*^2^)] = 0.055	Hydrogen site location: inferred from neighbouring sites
*wR*(*F*^2^) = 0.154	H-atom parameters constrained
*S* = 1.06	*w* = 1/[σ^2^(*F*_o_^2^) + (0.0765*P*)^2^ + 3.7684*P*] where *P* = (*F*_o_^2^ + 2*F*_c_^2^)/3
10145 reflections	(Δ/σ)_max_ = 0.001
661 parameters	Δρ_max_ = 0.51 e Å^−^^3^
0 restraints	Δρ_min_ = −0.62 e Å^−^^3^

### Special details

**Table:**

Experimental. CrysAlisPro (Agilent Technologies, 2012) Analytical numeric absorption correction using a multifaceted crystal model based on expressions derived by R.C. Clark & J.S. Reid. (Clark, R. C. & Reid, J. S. (1995). Acta Cryst. A51, 887-897)
Geometry. All e.s.d.'s (except the e.s.d. in the dihedral angle between two l.s. planes) are estimated using the full covariance matrix. The cell e.s.d.'s are taken into account individually in the estimation of e.s.d.'s in distances, angles and torsion angles; correlations between e.s.d.'s in cell parameters are only used when they are defined by crystal symmetry. An approximate (isotropic) treatment of cell e.s.d.'s is used for estimating e.s.d.'s involving l.s. planes.
Refinement. Refinement of *F*^2^ against ALL reflections. The weighted *R*-factor *wR* and goodness of fit *S* are based on *F*^2^, conventional *R*-factors *R* are based on *F*, with *F* set to zero for negative *F*^2^. The threshold expression of *F*^2^ > σ(*F*^2^) is used only for calculating *R*-factors(gt) *etc*. and is not relevant to the choice of reflections for refinement. *R*-factors based on *F*^2^ are statistically about twice as large as those based on *F*, and *R*- factors based on ALL data will be even larger.

### Fractional atomic coordinates and isotropic or equivalent isotropic displacement parameters (Å^2^)

**Table:**

	*x*	*y*	*z*	*U*_iso_*/*U*_eq_	
Co	0.38732 (3)	0.70424 (2)	0.356917 (14)	0.01928 (11)	
O1A	0.45006 (16)	0.79710 (9)	0.39056 (7)	0.0241 (4)	
O2A	0.49775 (14)	0.64526 (9)	0.40544 (6)	0.0219 (3)	
O3A	0.5460 (2)	1.00708 (13)	0.40380 (12)	0.0520 (6)	
C1A	0.5392 (2)	0.80635 (14)	0.42384 (10)	0.0226 (5)	
C2A	0.6100 (2)	0.74622 (14)	0.44765 (9)	0.0227 (5)	
C3A	0.7176 (2)	0.76390 (15)	0.47654 (10)	0.0246 (5)	
H3AA	0.7663	0.7233	0.4901	0.030*	
C4A	0.7548 (2)	0.83833 (15)	0.48580 (10)	0.0265 (5)	
C5A	0.6786 (2)	0.89697 (15)	0.46756 (10)	0.0282 (5)	
H5AA	0.7003	0.9483	0.4753	0.034*	
C6A	0.5727 (2)	0.88278 (14)	0.43856 (10)	0.0261 (5)	
C7A	0.8731 (2)	0.85516 (16)	0.51452 (11)	0.0316 (5)	
H7AA	0.9184	0.8078	0.5192	0.047*	
H7AB	0.8603	0.8775	0.5500	0.047*	
H7AC	0.9175	0.8912	0.4930	0.047*	
C8A	0.5713 (2)	0.66727 (14)	0.44132 (9)	0.0218 (4)	
C9A	0.6168 (2)	0.60673 (14)	0.47901 (9)	0.0223 (5)	
C10A	0.6254 (2)	0.53247 (14)	0.45905 (10)	0.0253 (5)	
H10A	0.6044	0.5222	0.4222	0.030*	
C11A	0.6646 (2)	0.47389 (14)	0.49288 (11)	0.0291 (5)	
H11A	0.6729	0.4239	0.4788	0.035*	
C12A	0.6918 (2)	0.48798 (16)	0.54746 (11)	0.0306 (5)	
H12A	0.7191	0.4477	0.5706	0.037*	
C13A	0.6790 (2)	0.56087 (16)	0.56794 (10)	0.0277 (5)	
H13A	0.6955	0.5702	0.6053	0.033*	
C14A	0.6423 (2)	0.62031 (15)	0.53404 (10)	0.0244 (5)	
H14A	0.6344	0.6703	0.5482	0.029*	
C15A	0.4986 (2)	0.94882 (15)	0.41900 (12)	0.0317 (5)	
C16A	0.3663 (2)	0.94498 (14)	0.42014 (11)	0.0282 (5)	
C17A	0.2985 (3)	0.98635 (16)	0.38188 (12)	0.0340 (6)	
H17A	0.3370	1.0149	0.3553	0.041*	
C18A	0.1754 (3)	0.98642 (17)	0.38207 (12)	0.0373 (6)	
H18A	0.1298	1.0135	0.3551	0.045*	
C19A	0.1197 (2)	0.94677 (17)	0.42181 (12)	0.0348 (6)	
H19A	0.0357	0.9474	0.4226	0.042*	
C20A	0.1863 (3)	0.90615 (16)	0.46045 (11)	0.0329 (6)	
H20A	0.1476	0.8794	0.4878	0.040*	
C21A	0.3092 (2)	0.90421 (15)	0.45952 (11)	0.0303 (5)	
H21A	0.3541	0.8752	0.4856	0.036*	
O1B	0.27825 (14)	0.76059 (10)	0.31121 (6)	0.0218 (3)	
O2B	0.26886 (15)	0.69853 (9)	0.41242 (7)	0.0222 (3)	
O3B	0.09769 (18)	0.80141 (14)	0.18133 (8)	0.0405 (5)	
C1B	0.1695 (2)	0.77906 (13)	0.31834 (9)	0.0197 (4)	
C2B	0.1057 (2)	0.76091 (13)	0.36543 (9)	0.0212 (4)	
C3B	−0.0114 (2)	0.78836 (14)	0.36943 (10)	0.0235 (5)	
H3BA	−0.0516	0.7780	0.4014	0.028*	
C4B	−0.0695 (2)	0.82958 (14)	0.32878 (10)	0.0257 (5)	
C5B	−0.0102 (2)	0.84059 (14)	0.28079 (10)	0.0256 (5)	
H5BA	−0.0511	0.8652	0.2512	0.031*	
C6B	0.1049 (2)	0.81700 (14)	0.27515 (9)	0.0230 (5)	
C7B	−0.1936 (2)	0.85986 (18)	0.33490 (12)	0.0339 (6)	
H7BA	−0.2464	0.8177	0.3435	0.051*	
H7BB	−0.2224	0.8839	0.3010	0.051*	
H7BC	−0.1925	0.8975	0.3641	0.051*	
C8B	0.1611 (2)	0.71668 (13)	0.40888 (9)	0.0202 (4)	
C9B	0.0913 (2)	0.68677 (14)	0.45423 (9)	0.0221 (4)	
C10B	−0.0060 (2)	0.63968 (15)	0.44322 (10)	0.0272 (5)	
H10B	−0.0347	0.6318	0.4069	0.033*	
C11B	−0.0611 (2)	0.60415 (17)	0.48537 (12)	0.0330 (6)	
H11B	−0.1253	0.5701	0.4779	0.040*	
C12B	−0.0223 (2)	0.61863 (19)	0.53844 (11)	0.0379 (7)	
H12B	−0.0614	0.5955	0.5673	0.045*	
C13B	0.0732 (2)	0.6666 (2)	0.54952 (11)	0.0366 (6)	
H13B	0.0984	0.6771	0.5859	0.044*	
C14B	0.1321 (2)	0.69935 (15)	0.50742 (10)	0.0279 (5)	
H14B	0.2000	0.7303	0.5149	0.034*	
C15B	0.1576 (2)	0.82364 (15)	0.22050 (10)	0.0264 (5)	
C16B	0.2749 (2)	0.86002 (15)	0.21381 (10)	0.0267 (5)	
C17B	0.3289 (2)	0.90612 (16)	0.25359 (11)	0.0321 (6)	
H17B	0.2956	0.9099	0.2879	0.039*	
C18B	0.4309 (3)	0.94661 (17)	0.24345 (13)	0.0387 (6)	
H18B	0.4663	0.9787	0.2706	0.046*	
C19B	0.4817 (3)	0.94033 (17)	0.19372 (13)	0.0372 (6)	
H19B	0.5509	0.9687	0.1866	0.045*	
C20B	0.4305 (2)	0.89232 (17)	0.15445 (12)	0.0345 (6)	
H20B	0.4663	0.8867	0.1208	0.041*	
C21B	0.3280 (2)	0.85279 (16)	0.16411 (11)	0.0296 (5)	
H21B	0.2932	0.8205	0.1369	0.035*	
O1C	0.32753 (15)	0.60991 (10)	0.32491 (6)	0.0229 (3)	
O2C	0.49974 (15)	0.70749 (10)	0.29840 (7)	0.0230 (3)	
O3C	0.16902 (17)	0.46986 (11)	0.31788 (8)	0.0331 (4)	
C1C	0.3010 (2)	0.60812 (13)	0.27299 (9)	0.0214 (4)	
C2C	0.3681 (2)	0.64691 (14)	0.23363 (10)	0.0241 (5)	
C3C	0.3266 (2)	0.64560 (15)	0.17862 (10)	0.0278 (5)	
H3CA	0.3704	0.6725	0.1529	0.033*	
C4C	0.2259 (3)	0.60711 (17)	0.16093 (10)	0.0317 (6)	
C5C	0.1635 (2)	0.56738 (16)	0.19942 (10)	0.0293 (5)	
H5CA	0.0942	0.5399	0.1879	0.035*	
C6C	0.1993 (2)	0.56678 (14)	0.25364 (10)	0.0238 (5)	
C7C	0.1833 (3)	0.6079 (2)	0.10236 (11)	0.0433 (7)	
H7CA	0.2346	0.6411	0.0820	0.065*	
H7CB	0.1016	0.6270	0.0992	0.065*	
H7CC	0.1858	0.5560	0.0878	0.065*	
C8C	0.4756 (2)	0.68717 (14)	0.25044 (10)	0.0232 (5)	
C9C	0.5663 (2)	0.70757 (14)	0.21124 (10)	0.0254 (5)	
C10C	0.6301 (2)	0.77469 (15)	0.22062 (10)	0.0277 (5)	
H10C	0.6129	0.8063	0.2504	0.033*	
C11C	0.7187 (3)	0.79563 (16)	0.18670 (12)	0.0338 (6)	
H11C	0.7617	0.8416	0.1931	0.041*	
C12C	0.7444 (3)	0.74934 (19)	0.14349 (13)	0.0413 (7)	
H12C	0.8059	0.7632	0.1206	0.050*	
C13C	0.6805 (3)	0.68285 (19)	0.13359 (13)	0.0422 (7)	
H13C	0.6976	0.6516	0.1036	0.051*	
C14C	0.5913 (3)	0.66184 (16)	0.16748 (12)	0.0343 (6)	
H14C	0.5476	0.6163	0.1607	0.041*	
C15C	0.1273 (2)	0.52477 (14)	0.29308 (10)	0.0244 (5)	
C16C	0.0036 (2)	0.55076 (14)	0.30089 (10)	0.0250 (5)	
C17C	−0.0408 (2)	0.61907 (15)	0.27878 (11)	0.0305 (5)	
H17C	0.0077	0.6498	0.2573	0.037*	
C18C	−0.1556 (3)	0.64190 (18)	0.28816 (14)	0.0397 (7)	
H18C	−0.1855	0.6882	0.2730	0.048*	
C19C	−0.2271 (3)	0.59739 (19)	0.31970 (14)	0.0417 (7)	
H19C	−0.3056	0.6133	0.3262	0.050*	
C20C	−0.1829 (3)	0.52935 (18)	0.34171 (13)	0.0390 (7)	
H20C	−0.2314	0.4988	0.3633	0.047*	
C21C	−0.0688 (2)	0.50623 (15)	0.33220 (11)	0.0301 (5)	
H21C	−0.0394	0.4596	0.3471	0.036*	

### Atomic displacement parameters (Å^2^)

**Table:**

	*U*^11^	*U*^22^	*U*^33^	*U*^12^	*U*^13^	*U*^23^
Co	0.0200 (2)	0.0188 (2)	0.01894 (19)	0.00065 (14)	0.00017 (13)	0.00137 (14)
O1A	0.0307 (9)	0.0180 (8)	0.0235 (8)	0.0033 (6)	−0.0002 (7)	0.0001 (6)
O2A	0.0243 (8)	0.0183 (8)	0.0228 (8)	−0.0010 (6)	−0.0029 (6)	0.0006 (6)
O3A	0.0395 (12)	0.0282 (11)	0.0885 (18)	−0.0021 (9)	0.0051 (11)	0.0192 (11)
C1A	0.0228 (11)	0.0203 (11)	0.0254 (11)	0.0004 (9)	0.0065 (9)	0.0006 (9)
C2A	0.0256 (11)	0.0199 (11)	0.0227 (11)	0.0001 (9)	0.0032 (8)	−0.0004 (9)
C3A	0.0237 (11)	0.0248 (12)	0.0255 (11)	0.0019 (9)	0.0028 (9)	−0.0005 (9)
C4A	0.0236 (11)	0.0279 (13)	0.0283 (12)	−0.0022 (10)	0.0057 (9)	−0.0039 (10)
C5A	0.0308 (13)	0.0221 (12)	0.0324 (13)	−0.0045 (10)	0.0080 (10)	−0.0049 (10)
C6A	0.0267 (12)	0.0216 (12)	0.0306 (12)	0.0018 (9)	0.0073 (9)	−0.0006 (9)
C7A	0.0289 (13)	0.0310 (13)	0.0351 (13)	−0.0049 (10)	0.0021 (10)	−0.0078 (11)
C8A	0.0196 (10)	0.0236 (12)	0.0225 (11)	0.0009 (9)	0.0041 (8)	0.0005 (9)
C9A	0.0197 (10)	0.0220 (11)	0.0254 (11)	−0.0006 (9)	0.0017 (8)	0.0022 (9)
C10A	0.0229 (11)	0.0230 (12)	0.0302 (12)	−0.0002 (9)	0.0022 (9)	−0.0001 (9)
C11A	0.0271 (12)	0.0179 (11)	0.0424 (14)	0.0010 (9)	0.0033 (10)	0.0030 (10)
C12A	0.0261 (12)	0.0279 (13)	0.0378 (14)	−0.0006 (10)	0.0014 (10)	0.0142 (11)
C13A	0.0227 (11)	0.0354 (14)	0.0250 (11)	−0.0021 (10)	0.0001 (9)	0.0057 (10)
C14A	0.0234 (11)	0.0251 (12)	0.0248 (11)	0.0000 (9)	0.0022 (9)	0.0012 (9)
C15A	0.0345 (14)	0.0186 (12)	0.0422 (14)	0.0000 (10)	0.0052 (11)	0.0010 (10)
C16A	0.0329 (13)	0.0174 (11)	0.0345 (13)	0.0002 (10)	0.0025 (10)	−0.0046 (10)
C17A	0.0398 (15)	0.0253 (13)	0.0369 (14)	0.0027 (11)	0.0029 (11)	0.0014 (11)
C18A	0.0386 (15)	0.0316 (14)	0.0412 (15)	0.0050 (12)	−0.0039 (12)	0.0014 (12)
C19A	0.0276 (13)	0.0315 (14)	0.0453 (15)	0.0009 (11)	0.0014 (11)	−0.0084 (12)
C20A	0.0353 (14)	0.0304 (14)	0.0336 (13)	−0.0008 (11)	0.0064 (11)	−0.0043 (11)
C21A	0.0338 (13)	0.0237 (12)	0.0336 (13)	0.0011 (10)	0.0027 (10)	−0.0031 (10)
O1B	0.0217 (8)	0.0233 (8)	0.0205 (7)	0.0031 (6)	0.0024 (6)	0.0048 (6)
O2B	0.0236 (8)	0.0235 (8)	0.0194 (8)	0.0016 (6)	0.0011 (6)	0.0034 (6)
O3B	0.0351 (11)	0.0653 (15)	0.0208 (9)	−0.0087 (10)	−0.0006 (7)	0.0042 (9)
C1B	0.0215 (11)	0.0163 (10)	0.0211 (10)	0.0000 (8)	−0.0002 (8)	0.0008 (8)
C2B	0.0240 (11)	0.0191 (11)	0.0206 (10)	−0.0010 (9)	0.0013 (8)	−0.0007 (8)
C3B	0.0256 (12)	0.0245 (12)	0.0208 (11)	0.0003 (9)	0.0039 (9)	−0.0008 (9)
C4B	0.0241 (12)	0.0230 (12)	0.0299 (12)	0.0014 (9)	0.0004 (9)	0.0020 (9)
C5B	0.0243 (11)	0.0246 (12)	0.0274 (12)	0.0028 (9)	−0.0030 (9)	0.0054 (9)
C6B	0.0257 (12)	0.0207 (11)	0.0226 (11)	−0.0010 (9)	0.0002 (9)	0.0027 (9)
C7B	0.0259 (12)	0.0411 (15)	0.0349 (14)	0.0081 (11)	0.0034 (10)	0.0052 (12)
C8B	0.0237 (11)	0.0179 (10)	0.0192 (10)	−0.0016 (8)	0.0024 (8)	−0.0017 (8)
C9B	0.0241 (11)	0.0212 (11)	0.0212 (11)	0.0020 (9)	0.0029 (8)	0.0030 (9)
C10B	0.0276 (12)	0.0283 (12)	0.0256 (12)	−0.0003 (10)	0.0005 (9)	0.0026 (10)
C11B	0.0239 (12)	0.0346 (14)	0.0407 (14)	−0.0033 (10)	0.0034 (10)	0.0106 (12)
C12B	0.0274 (13)	0.0556 (19)	0.0313 (13)	0.0029 (12)	0.0067 (10)	0.0189 (13)
C13B	0.0306 (13)	0.0591 (19)	0.0201 (11)	0.0046 (13)	0.0016 (10)	0.0069 (12)
C14B	0.0268 (12)	0.0340 (14)	0.0230 (12)	−0.0009 (10)	0.0005 (9)	0.0009 (10)
C15B	0.0282 (12)	0.0268 (12)	0.0241 (12)	0.0036 (10)	−0.0001 (9)	0.0071 (9)
C16B	0.0299 (12)	0.0249 (12)	0.0252 (11)	0.0036 (10)	0.0018 (9)	0.0101 (9)
C17B	0.0340 (14)	0.0298 (13)	0.0330 (13)	−0.0019 (11)	0.0060 (10)	0.0049 (11)
C18B	0.0396 (15)	0.0323 (15)	0.0445 (16)	−0.0046 (12)	0.0044 (12)	0.0008 (12)
C19B	0.0308 (14)	0.0308 (14)	0.0507 (17)	−0.0026 (11)	0.0099 (12)	0.0103 (12)
C20B	0.0326 (13)	0.0368 (15)	0.0350 (14)	0.0075 (11)	0.0103 (11)	0.0121 (11)
C21B	0.0294 (12)	0.0317 (13)	0.0277 (12)	0.0062 (10)	0.0029 (10)	0.0087 (10)
O1C	0.0256 (8)	0.0210 (8)	0.0219 (8)	−0.0029 (6)	−0.0016 (6)	−0.0004 (6)
O2C	0.0221 (8)	0.0244 (9)	0.0226 (8)	0.0004 (6)	0.0018 (6)	0.0005 (6)
O3C	0.0320 (10)	0.0247 (9)	0.0420 (10)	0.0010 (8)	−0.0037 (8)	0.0058 (8)
C1C	0.0210 (11)	0.0205 (11)	0.0226 (11)	0.0006 (9)	−0.0004 (8)	−0.0011 (9)
C2C	0.0262 (11)	0.0213 (11)	0.0249 (11)	0.0021 (9)	0.0018 (9)	−0.0013 (9)
C3C	0.0324 (13)	0.0288 (13)	0.0226 (11)	0.0015 (10)	0.0036 (9)	−0.0001 (9)
C4C	0.0374 (14)	0.0350 (14)	0.0223 (11)	0.0018 (11)	−0.0033 (10)	−0.0015 (10)
C5C	0.0285 (12)	0.0302 (13)	0.0289 (12)	−0.0031 (10)	−0.0032 (9)	−0.0053 (10)
C6C	0.0226 (11)	0.0216 (11)	0.0269 (11)	0.0002 (9)	0.0002 (9)	−0.0013 (9)
C7C	0.0485 (17)	0.0557 (19)	0.0249 (13)	−0.0068 (15)	−0.0050 (12)	0.0002 (13)
C8C	0.0251 (12)	0.0187 (11)	0.0259 (11)	0.0047 (9)	0.0035 (9)	0.0015 (9)
C9C	0.0252 (12)	0.0239 (12)	0.0273 (12)	0.0025 (9)	0.0040 (9)	0.0028 (9)
C10C	0.0298 (12)	0.0249 (12)	0.0282 (12)	−0.0001 (10)	−0.0019 (9)	0.0037 (10)
C11C	0.0311 (14)	0.0313 (14)	0.0389 (15)	−0.0044 (11)	−0.0007 (11)	0.0088 (11)
C12C	0.0408 (16)	0.0409 (16)	0.0437 (16)	−0.0028 (13)	0.0172 (12)	0.0117 (13)
C13C	0.0497 (18)	0.0371 (16)	0.0420 (16)	0.0002 (14)	0.0234 (14)	−0.0006 (13)
C14C	0.0384 (15)	0.0276 (13)	0.0380 (14)	−0.0024 (11)	0.0135 (11)	−0.0014 (11)
C15C	0.0253 (11)	0.0204 (11)	0.0271 (11)	−0.0028 (9)	−0.0032 (9)	−0.0031 (9)
C16C	0.0240 (11)	0.0220 (11)	0.0287 (12)	−0.0027 (9)	−0.0025 (9)	−0.0031 (9)
C17C	0.0284 (12)	0.0244 (12)	0.0380 (14)	0.0003 (10)	−0.0032 (10)	−0.0006 (10)
C18C	0.0306 (14)	0.0328 (15)	0.0550 (18)	0.0074 (11)	−0.0058 (12)	−0.0044 (13)
C19C	0.0243 (13)	0.0443 (17)	0.0567 (18)	−0.0012 (12)	0.0039 (12)	−0.0182 (14)
C20C	0.0339 (14)	0.0375 (16)	0.0462 (16)	−0.0108 (12)	0.0096 (12)	−0.0122 (13)
C21C	0.0304 (13)	0.0256 (12)	0.0343 (13)	−0.0076 (10)	0.0032 (10)	−0.0033 (10)

### Geometric parameters (Å, º)

**Table:**

Co—O1B	1.9063 (16)	C9B—C10B	1.390 (4)
Co—O1C	1.9414 (17)	C9B—C14B	1.391 (3)
Co—O1A	1.9470 (17)	C10B—C11B	1.390 (4)
Co—O2B	1.9682 (17)	C10B—H10B	0.9500
Co—O2C	1.9749 (17)	C11B—C12B	1.388 (4)
Co—O2A	1.9793 (16)	C11B—H11B	0.9500
O1A—C1A	1.279 (3)	C12B—C13B	1.383 (4)
O2A—C8A	1.247 (3)	C12B—H12B	0.9500
O3A—C15A	1.222 (3)	C13B—C14B	1.389 (4)
C1A—C2A	1.432 (3)	C13B—H13B	0.9500
C1A—C6A	1.435 (3)	C14B—H14B	0.9500
C2A—C3A	1.413 (3)	C15B—C16B	1.487 (4)
C2A—C8A	1.459 (3)	C16B—C17B	1.391 (4)
C3A—C4A	1.387 (4)	C16B—C21B	1.402 (4)
C3A—H3AA	0.9500	C17B—C18B	1.387 (4)
C4A—C5A	1.401 (4)	C17B—H17B	0.9500
C4A—C7A	1.510 (3)	C18B—C19B	1.389 (4)
C5A—C6A	1.386 (4)	C18B—H18B	0.9500
C5A—H5AA	0.9500	C19B—C20B	1.390 (4)
C6A—C15A	1.495 (4)	C19B—H19B	0.9500
C7A—H7AA	0.9800	C20B—C21B	1.380 (4)
C7A—H7AB	0.9800	C20B—H20B	0.9500
C7A—H7AC	0.9800	C21B—H21B	0.9500
C8A—C9A	1.488 (3)	O1C—C1C	1.306 (3)
C9A—C10A	1.399 (3)	O2C—C8C	1.257 (3)
C9A—C14A	1.399 (3)	O3C—C15C	1.224 (3)
C10A—C11A	1.384 (4)	C1C—C6C	1.421 (3)
C10A—H10A	0.9500	C1C—C2C	1.436 (3)
C11A—C12A	1.394 (4)	C2C—C3C	1.418 (3)
C11A—H11A	0.9500	C2C—C8C	1.447 (3)
C12A—C13A	1.386 (4)	C3C—C4C	1.375 (4)
C12A—H12A	0.9500	C3C—H3CA	0.9500
C13A—C14A	1.389 (4)	C4C—C5C	1.400 (4)
C13A—H13A	0.9500	C4C—C7C	1.506 (4)
C14A—H14A	0.9500	C5C—C6C	1.383 (3)
C15A—C16A	1.497 (4)	C5C—H5CA	0.9500
C16A—C17A	1.392 (4)	C6C—C15C	1.496 (3)
C16A—C21A	1.394 (4)	C7C—H7CA	0.9800
C17A—C18A	1.390 (4)	C7C—H7CB	0.9800
C17A—H17A	0.9500	C7C—H7CC	0.9800
C18A—C19A	1.382 (4)	C8C—C9C	1.490 (3)
C18A—H18A	0.9500	C9C—C14C	1.389 (4)
C19A—C20A	1.384 (4)	C9C—C10C	1.394 (4)
C19A—H19A	0.9500	C10C—C11C	1.388 (4)
C20A—C21A	1.388 (4)	C10C—H10C	0.9500
C20A—H20A	0.9500	C11C—C12C	1.386 (5)
C21A—H21A	0.9500	C11C—H11C	0.9500
O1B—C1B	1.290 (3)	C12C—C13C	1.387 (5)
O2B—C8B	1.256 (3)	C12C—H12C	0.9500
O3B—C15B	1.219 (3)	C13C—C14C	1.392 (4)
C1B—C6B	1.429 (3)	C13C—H13C	0.9500
C1B—C2B	1.437 (3)	C14C—H14C	0.9500
C2B—C3B	1.415 (3)	C15C—C16C	1.491 (3)
C2B—C8B	1.443 (3)	C16C—C21C	1.394 (4)
C3B—C4B	1.379 (3)	C16C—C17C	1.400 (4)
C3B—H3BA	0.9500	C17C—C18C	1.387 (4)
C4B—C5B	1.407 (3)	C17C—H17C	0.9500
C4B—C7B	1.513 (3)	C18C—C19C	1.391 (5)
C5B—C6B	1.377 (3)	C18C—H18C	0.9500
C5B—H5BA	0.9500	C19C—C20C	1.394 (5)
C6B—C15B	1.511 (3)	C19C—H19C	0.9500
C7B—H7BA	0.9800	C20C—C21C	1.382 (4)
C7B—H7BB	0.9800	C20C—H20C	0.9500
C7B—H7BC	0.9800	C21C—H21C	0.9500
C8B—C9B	1.499 (3)		
			
O1B—Co—O1C	89.93 (7)	O2B—C8B—C9B	113.7 (2)
O1B—Co—O1A	91.73 (7)	C2B—C8B—C9B	121.5 (2)
O1C—Co—O1A	178.32 (7)	C10B—C9B—C14B	120.0 (2)
O1B—Co—O2B	90.02 (7)	C10B—C9B—C8B	120.0 (2)
O1C—Co—O2B	90.44 (7)	C14B—C9B—C8B	119.5 (2)
O1A—Co—O2B	89.35 (7)	C11B—C10B—C9B	119.9 (2)
O1B—Co—O2C	88.19 (7)	C11B—C10B—H10B	120.1
O1C—Co—O2C	87.01 (7)	C9B—C10B—H10B	120.1
O1A—Co—O2C	93.25 (7)	C12B—C11B—C10B	119.8 (3)
O2B—Co—O2C	176.88 (7)	C12B—C11B—H11B	120.1
O1B—Co—O2A	178.76 (7)	C10B—C11B—H11B	120.1
O1C—Co—O2A	89.87 (7)	C13B—C12B—C11B	120.3 (2)
O1A—Co—O2A	88.47 (7)	C13B—C12B—H12B	119.8
O2B—Co—O2A	88.75 (7)	C11B—C12B—H12B	119.8
O2C—Co—O2A	93.02 (7)	C12B—C13B—C14B	120.0 (3)
C1A—O1A—Co	129.76 (16)	C12B—C13B—H13B	120.0
C8A—O2A—Co	130.35 (16)	C14B—C13B—H13B	120.0
O1A—C1A—C2A	125.2 (2)	C13B—C14B—C9B	119.8 (2)
O1A—C1A—C6A	118.1 (2)	C13B—C14B—H14B	120.1
C2A—C1A—C6A	116.7 (2)	C9B—C14B—H14B	120.1
C3A—C2A—C1A	119.6 (2)	O3B—C15B—C16B	120.7 (2)
C3A—C2A—C8A	120.6 (2)	O3B—C15B—C6B	117.5 (2)
C1A—C2A—C8A	119.8 (2)	C16B—C15B—C6B	121.8 (2)
C4A—C3A—C2A	122.4 (2)	C17B—C16B—C21B	118.9 (2)
C4A—C3A—H3AA	118.8	C17B—C16B—C15B	122.1 (2)
C2A—C3A—H3AA	118.8	C21B—C16B—C15B	118.8 (2)
C3A—C4A—C5A	117.5 (2)	C18B—C17B—C16B	120.5 (3)
C3A—C4A—C7A	121.0 (2)	C18B—C17B—H17B	119.8
C5A—C4A—C7A	121.5 (2)	C16B—C17B—H17B	119.8
C6A—C5A—C4A	122.3 (2)	C17B—C18B—C19B	120.2 (3)
C6A—C5A—H5AA	118.8	C17B—C18B—H18B	119.9
C4A—C5A—H5AA	118.8	C19B—C18B—H18B	119.9
C5A—C6A—C1A	120.5 (2)	C18B—C19B—C20B	119.6 (3)
C5A—C6A—C15A	118.8 (2)	C18B—C19B—H19B	120.2
C1A—C6A—C15A	120.4 (2)	C20B—C19B—H19B	120.2
C4A—C7A—H7AA	109.5	C21B—C20B—C19B	120.3 (3)
C4A—C7A—H7AB	109.5	C21B—C20B—H20B	119.8
H7AA—C7A—H7AB	109.5	C19B—C20B—H20B	119.8
C4A—C7A—H7AC	109.5	C20B—C21B—C16B	120.4 (3)
H7AA—C7A—H7AC	109.5	C20B—C21B—H21B	119.8
H7AB—C7A—H7AC	109.5	C16B—C21B—H21B	119.8
O2A—C8A—C2A	123.8 (2)	C1C—O1C—Co	118.70 (15)
O2A—C8A—C9A	115.0 (2)	C8C—O2C—Co	124.66 (16)
C2A—C8A—C9A	121.2 (2)	O1C—C1C—C6C	119.0 (2)
C10A—C9A—C14A	119.2 (2)	O1C—C1C—C2C	123.7 (2)
C10A—C9A—C8A	118.1 (2)	C6C—C1C—C2C	117.2 (2)
C14A—C9A—C8A	122.5 (2)	C3C—C2C—C1C	119.0 (2)
C11A—C10A—C9A	120.2 (2)	C3C—C2C—C8C	121.0 (2)
C11A—C10A—H10A	119.9	C1C—C2C—C8C	119.9 (2)
C9A—C10A—H10A	119.9	C4C—C3C—C2C	122.8 (2)
C10A—C11A—C12A	120.3 (2)	C4C—C3C—H3CA	118.6
C10A—C11A—H11A	119.9	C2C—C3C—H3CA	118.6
C12A—C11A—H11A	119.9	C3C—C4C—C5C	117.6 (2)
C13A—C12A—C11A	119.8 (2)	C3C—C4C—C7C	121.5 (3)
C13A—C12A—H12A	120.1	C5C—C4C—C7C	120.9 (3)
C11A—C12A—H12A	120.1	C6C—C5C—C4C	122.2 (2)
C12A—C13A—C14A	120.3 (2)	C6C—C5C—H5CA	118.9
C12A—C13A—H13A	119.8	C4C—C5C—H5CA	118.9
C14A—C13A—H13A	119.8	C5C—C6C—C1C	121.0 (2)
C13A—C14A—C9A	120.1 (2)	C5C—C6C—C15C	119.8 (2)
C13A—C14A—H14A	119.9	C1C—C6C—C15C	119.2 (2)
C9A—C14A—H14A	119.9	C4C—C7C—H7CA	109.5
O3A—C15A—C6A	120.2 (3)	C4C—C7C—H7CB	109.5
O3A—C15A—C16A	119.7 (3)	H7CA—C7C—H7CB	109.5
C6A—C15A—C16A	120.1 (2)	C4C—C7C—H7CC	109.5
C17A—C16A—C21A	119.1 (3)	H7CA—C7C—H7CC	109.5
C17A—C16A—C15A	118.6 (2)	H7CB—C7C—H7CC	109.5
C21A—C16A—C15A	122.3 (2)	O2C—C8C—C2C	123.3 (2)
C18A—C17A—C16A	120.9 (3)	O2C—C8C—C9C	115.1 (2)
C18A—C17A—H17A	119.6	C2C—C8C—C9C	121.6 (2)
C16A—C17A—H17A	119.6	C14C—C9C—C10C	119.6 (2)
C19A—C18A—C17A	119.5 (3)	C14C—C9C—C8C	122.9 (2)
C19A—C18A—H18A	120.3	C10C—C9C—C8C	117.4 (2)
C17A—C18A—H18A	120.3	C11C—C10C—C9C	120.3 (3)
C18A—C19A—C20A	120.1 (3)	C11C—C10C—H10C	119.8
C18A—C19A—H19A	120.0	C9C—C10C—H10C	119.8
C20A—C19A—H19A	120.0	C12C—C11C—C10C	119.8 (3)
C19A—C20A—C21A	120.6 (3)	C12C—C11C—H11C	120.1
C19A—C20A—H20A	119.7	C10C—C11C—H11C	120.1
C21A—C20A—H20A	119.7	C11C—C12C—C13C	120.2 (3)
C20A—C21A—C16A	119.8 (3)	C11C—C12C—H12C	119.9
C20A—C21A—H21A	120.1	C13C—C12C—H12C	119.9
C16A—C21A—H21A	120.1	C12C—C13C—C14C	120.1 (3)
C1B—O1B—Co	129.72 (14)	C12C—C13C—H13C	120.0
C8B—O2B—Co	128.88 (15)	C14C—C13C—H13C	120.0
O1B—C1B—C6B	117.9 (2)	C9C—C14C—C13C	120.0 (3)
O1B—C1B—C2B	125.1 (2)	C9C—C14C—H14C	120.0
C6B—C1B—C2B	117.0 (2)	C13C—C14C—H14C	120.0
C3B—C2B—C1B	119.3 (2)	O3C—C15C—C16C	121.0 (2)
C3B—C2B—C8B	120.1 (2)	O3C—C15C—C6C	120.5 (2)
C1B—C2B—C8B	120.6 (2)	C16C—C15C—C6C	118.5 (2)
C4B—C3B—C2B	122.6 (2)	C21C—C16C—C17C	119.3 (2)
C4B—C3B—H3BA	118.7	C21C—C16C—C15C	118.7 (2)
C2B—C3B—H3BA	118.7	C17C—C16C—C15C	122.0 (2)
C3B—C4B—C5B	117.4 (2)	C18C—C17C—C16C	120.1 (3)
C3B—C4B—C7B	121.3 (2)	C18C—C17C—H17C	120.0
C5B—C4B—C7B	121.3 (2)	C16C—C17C—H17C	120.0
C6B—C5B—C4B	122.4 (2)	C17C—C18C—C19C	120.3 (3)
C6B—C5B—H5BA	118.8	C17C—C18C—H18C	119.8
C4B—C5B—H5BA	118.8	C19C—C18C—H18C	119.8
C5B—C6B—C1B	120.8 (2)	C18C—C19C—C20C	119.6 (3)
C5B—C6B—C15B	118.9 (2)	C18C—C19C—H19C	120.2
C1B—C6B—C15B	119.9 (2)	C20C—C19C—H19C	120.2
C4B—C7B—H7BA	109.5	C21C—C20C—C19C	120.2 (3)
C4B—C7B—H7BB	109.5	C21C—C20C—H20C	119.9
H7BA—C7B—H7BB	109.5	C19C—C20C—H20C	119.9
C4B—C7B—H7BC	109.5	C20C—C21C—C16C	120.5 (3)
H7BA—C7B—H7BC	109.5	C20C—C21C—H21C	119.7
H7BB—C7B—H7BC	109.5	C16C—C21C—H21C	119.7
O2B—C8B—C2B	124.7 (2)		
			
O1B—Co—O1A—C1A	−172.3 (2)	C3B—C2B—C8B—C9B	11.1 (3)
O2B—Co—O1A—C1A	97.7 (2)	C1B—C2B—C8B—C9B	−170.4 (2)
O2C—Co—O1A—C1A	−84.0 (2)	O2B—C8B—C9B—C10B	−121.5 (2)
O2A—Co—O1A—C1A	9.0 (2)	C2B—C8B—C9B—C10B	57.5 (3)
O1C—Co—O2A—C8A	−179.2 (2)	O2B—C8B—C9B—C14B	51.4 (3)
O1A—Co—O2A—C8A	0.7 (2)	C2B—C8B—C9B—C14B	−129.7 (3)
O2B—Co—O2A—C8A	−88.7 (2)	C14B—C9B—C10B—C11B	−1.1 (4)
O2C—Co—O2A—C8A	93.8 (2)	C8B—C9B—C10B—C11B	171.7 (2)
Co—O1A—C1A—C2A	−5.2 (3)	C9B—C10B—C11B—C12B	3.0 (4)
Co—O1A—C1A—C6A	174.29 (16)	C10B—C11B—C12B—C13B	−1.8 (5)
O1A—C1A—C2A—C3A	169.3 (2)	C11B—C12B—C13B—C14B	−1.3 (5)
C6A—C1A—C2A—C3A	−10.1 (3)	C12B—C13B—C14B—C9B	3.1 (4)
O1A—C1A—C2A—C8A	−9.9 (4)	C10B—C9B—C14B—C13B	−1.9 (4)
C6A—C1A—C2A—C8A	170.6 (2)	C8B—C9B—C14B—C13B	−174.8 (2)
C1A—C2A—C3A—C4A	4.1 (4)	C5B—C6B—C15B—O3B	−46.5 (4)
C8A—C2A—C3A—C4A	−176.7 (2)	C1B—C6B—C15B—O3B	126.7 (3)
C2A—C3A—C4A—C5A	3.0 (4)	C5B—C6B—C15B—C16B	129.9 (3)
C2A—C3A—C4A—C7A	−177.0 (2)	C1B—C6B—C15B—C16B	−56.9 (3)
C3A—C4A—C5A—C6A	−3.6 (4)	O3B—C15B—C16B—C17B	160.0 (3)
C7A—C4A—C5A—C6A	176.4 (2)	C6B—C15B—C16B—C17B	−16.3 (4)
C4A—C5A—C6A—C1A	−2.9 (4)	O3B—C15B—C16B—C21B	−14.6 (4)
C4A—C5A—C6A—C15A	−177.9 (2)	C6B—C15B—C16B—C21B	169.1 (2)
O1A—C1A—C6A—C5A	−169.9 (2)	C21B—C16B—C17B—C18B	2.5 (4)
C2A—C1A—C6A—C5A	9.6 (3)	C15B—C16B—C17B—C18B	−172.0 (3)
O1A—C1A—C6A—C15A	5.1 (3)	C16B—C17B—C18B—C19B	−1.3 (4)
C2A—C1A—C6A—C15A	−175.4 (2)	C17B—C18B—C19B—C20B	−1.0 (5)
Co—O2A—C8A—C2A	−13.9 (3)	C18B—C19B—C20B—C21B	1.9 (4)
Co—O2A—C8A—C9A	165.00 (15)	C19B—C20B—C21B—C16B	−0.6 (4)
C3A—C2A—C8A—O2A	−159.7 (2)	C17B—C16B—C21B—C20B	−1.6 (4)
C1A—C2A—C8A—O2A	19.6 (3)	C15B—C16B—C21B—C20B	173.2 (2)
C3A—C2A—C8A—C9A	21.5 (3)	O1B—Co—O1C—C1C	39.09 (17)
C1A—C2A—C8A—C9A	−159.2 (2)	O2B—Co—O1C—C1C	129.11 (17)
O2A—C8A—C9A—C10A	31.2 (3)	O2C—Co—O1C—C1C	−49.11 (17)
C2A—C8A—C9A—C10A	−149.9 (2)	O2A—Co—O1C—C1C	−142.14 (17)
O2A—C8A—C9A—C14A	−143.8 (2)	O1B—Co—O2C—C8C	−52.61 (19)
C2A—C8A—C9A—C14A	35.1 (3)	O1C—Co—O2C—C8C	37.41 (19)
C14A—C9A—C10A—C11A	−3.3 (4)	O1A—Co—O2C—C8C	−144.25 (19)
C8A—C9A—C10A—C11A	−178.5 (2)	O2A—Co—O2C—C8C	127.12 (19)
C9A—C10A—C11A—C12A	2.2 (4)	Co—O1C—C1C—C6C	−141.27 (18)
C10A—C11A—C12A—C13A	0.4 (4)	Co—O1C—C1C—C2C	37.7 (3)
C11A—C12A—C13A—C14A	−1.8 (4)	O1C—C1C—C2C—C3C	−175.6 (2)
C12A—C13A—C14A—C9A	0.6 (4)	C6C—C1C—C2C—C3C	3.4 (3)
C10A—C9A—C14A—C13A	1.9 (4)	O1C—C1C—C2C—C8C	3.8 (4)
C8A—C9A—C14A—C13A	176.8 (2)	C6C—C1C—C2C—C8C	−177.2 (2)
C5A—C6A—C15A—O3A	36.1 (4)	C1C—C2C—C3C—C4C	−1.6 (4)
C1A—C6A—C15A—O3A	−139.0 (3)	C8C—C2C—C3C—C4C	179.0 (2)
C5A—C6A—C15A—C16A	−141.4 (3)	C2C—C3C—C4C—C5C	−0.5 (4)
C1A—C6A—C15A—C16A	43.6 (4)	C2C—C3C—C4C—C7C	179.0 (3)
O3A—C15A—C16A—C17A	31.6 (4)	C3C—C4C—C5C—C6C	0.8 (4)
C6A—C15A—C16A—C17A	−150.9 (3)	C7C—C4C—C5C—C6C	−178.8 (3)
O3A—C15A—C16A—C21A	−145.5 (3)	C4C—C5C—C6C—C1C	1.2 (4)
C6A—C15A—C16A—C21A	31.9 (4)	C4C—C5C—C6C—C15C	178.6 (2)
C21A—C16A—C17A—C18A	−1.0 (4)	O1C—C1C—C6C—C5C	175.8 (2)
C15A—C16A—C17A—C18A	−178.2 (3)	C2C—C1C—C6C—C5C	−3.2 (4)
C16A—C17A—C18A—C19A	2.0 (4)	O1C—C1C—C6C—C15C	−1.6 (3)
C17A—C18A—C19A—C20A	−1.3 (4)	C2C—C1C—C6C—C15C	179.4 (2)
C18A—C19A—C20A—C21A	−0.5 (4)	Co—O2C—C8C—C2C	−10.6 (3)
C19A—C20A—C21A—C16A	1.6 (4)	Co—O2C—C8C—C9C	169.42 (15)
C17A—C16A—C21A—C20A	−0.8 (4)	C3C—C2C—C8C—O2C	160.4 (2)
C15A—C16A—C21A—C20A	176.3 (2)	C1C—C2C—C8C—O2C	−18.9 (4)
O1C—Co—O1B—C1B	86.8 (2)	C3C—C2C—C8C—C9C	−19.5 (4)
O1A—Co—O1B—C1B	−93.0 (2)	C1C—C2C—C8C—C9C	161.1 (2)
O2B—Co—O1B—C1B	−3.6 (2)	O2C—C8C—C9C—C14C	145.7 (3)
O2C—Co—O1B—C1B	173.8 (2)	C2C—C8C—C9C—C14C	−34.3 (4)
O1B—Co—O2B—C8B	9.7 (2)	O2C—C8C—C9C—C10C	−32.4 (3)
O1C—Co—O2B—C8B	−80.2 (2)	C2C—C8C—C9C—C10C	147.5 (2)
O1A—Co—O2B—C8B	101.5 (2)	C14C—C9C—C10C—C11C	−0.4 (4)
O2A—Co—O2B—C8B	−170.1 (2)	C8C—C9C—C10C—C11C	177.8 (2)
Co—O1B—C1B—C6B	−175.28 (16)	C9C—C10C—C11C—C12C	−0.4 (4)
Co—O1B—C1B—C2B	1.2 (3)	C10C—C11C—C12C—C13C	1.1 (5)
O1B—C1B—C2B—C3B	176.5 (2)	C11C—C12C—C13C—C14C	−0.9 (5)
C6B—C1B—C2B—C3B	−7.0 (3)	C10C—C9C—C14C—C13C	0.6 (4)
O1B—C1B—C2B—C8B	−2.1 (4)	C8C—C9C—C14C—C13C	−177.5 (3)
C6B—C1B—C2B—C8B	174.5 (2)	C12C—C13C—C14C—C9C	0.0 (5)
C1B—C2B—C3B—C4B	2.8 (4)	C5C—C6C—C15C—O3C	116.0 (3)
C8B—C2B—C3B—C4B	−178.7 (2)	C1C—C6C—C15C—O3C	−66.6 (3)
C2B—C3B—C4B—C5B	3.3 (4)	C5C—C6C—C15C—C16C	−63.3 (3)
C2B—C3B—C4B—C7B	−178.4 (2)	C1C—C6C—C15C—C16C	114.1 (3)
C3B—C4B—C5B—C6B	−5.1 (4)	O3C—C15C—C16C—C21C	−7.4 (4)
C7B—C4B—C5B—C6B	176.6 (3)	C6C—C15C—C16C—C21C	171.9 (2)
C4B—C5B—C6B—C1B	0.6 (4)	O3C—C15C—C16C—C17C	171.2 (2)
C4B—C5B—C6B—C15B	173.7 (2)	C6C—C15C—C16C—C17C	−9.5 (3)
O1B—C1B—C6B—C5B	−177.8 (2)	C21C—C16C—C17C—C18C	0.1 (4)
C2B—C1B—C6B—C5B	5.4 (3)	C15C—C16C—C17C—C18C	−178.5 (2)
O1B—C1B—C6B—C15B	9.2 (3)	C16C—C17C—C18C—C19C	0.3 (4)
C2B—C1B—C6B—C15B	−167.6 (2)	C17C—C18C—C19C—C20C	−0.3 (5)
Co—O2B—C8B—C2B	−13.4 (3)	C18C—C19C—C20C—C21C	−0.1 (5)
Co—O2B—C8B—C9B	165.51 (15)	C19C—C20C—C21C—C16C	0.4 (4)
C3B—C2B—C8B—O2B	−170.1 (2)	C17C—C16C—C21C—C20C	−0.4 (4)
C1B—C2B—C8B—O2B	8.4 (4)	C15C—C16C—C21C—C20C	178.2 (2)

### Hydrogen-bond geometry (Å, º)

Cg7, Cg9, Cg11 and Cg12 are the centroids of the C9*A*–C14*A*, C9*C*–C14*C*, C16*B*–C21*B* and C16*C*–C21*C* rings, respectively.

**Table:**

*D*—H···*A*	*D*—H	H···*A*	*D*···*A*	*D*—H···*A*
C12*A*—H12*A*···O2*B*^i^	0.95	2.60	3.442 (3)	147
C13*A*—H13*A*···O3*C*^i^	0.95	2.48	3.278 (3)	141
C13*B*—H13*B*···O3*B*^ii^	0.95	2.39	3.311 (3)	162
C11*C*—H11*C*···O3*C*^iii^	0.95	2.40	3.313 (3)	161
C10*B*—H10*B*···*Cg*12	0.95	2.70	3.634 (3)	166
C11*B*—H11*B*···*Cg*7^iv^	0.95	2.72	3.479 (3)	137
C18*C*—H18*C*···*Cg*9^iv^	0.95	2.99	3.720 (4)	135
C20*C*—H20*C*···*Cg*11^v^	0.95	2.88	3.332 (3)	110

Symmetry codes: (i) −*x*+1, −*y*+1, −*z*+1; (ii) *x*, −*y*+3/2, *z*+1/2; (iii) −*x*+1, *y*+1/2, −*z*+1/2; (iv) *x*+1, *y*, *z*; (v) −*x*+2, *y*−1/2, −*z*+1/2.
